# Navigating the Confluence of Esophageal Disruption and Pyopneumothorax: A Complex Clinical Encounter

**DOI:** 10.7759/cureus.64661

**Published:** 2024-07-16

**Authors:** Jay Bhanushali, Ulhas Jadhav, Pankaj Wagh, Arman Sindhu, Bingu Shiv Kiran Reddy

**Affiliations:** 1 Respiratory Medicine, Datta Meghe Institute of Higher Education and Research, Wardha, IND; 2 Pulmonary and Critical Care Medicine, Datta Meghe Institute of Higher Education and Research, Wardha, IND

**Keywords:** oral contrast, fistula, esophageal cancer, pleura, empyema

## Abstract

Esophagopleural fistula (EPF) is a rare complication often associated with underlying esophageal malignancies. We present the case of a 64-year-old male who presented with left-sided pyopneumothorax and was diagnosed with EPF secondary to esophageal carcinoma. Imaging studies revealed a hydropneumothorax with an esophageal-pulmonary fistula communicating with the pleural cavity. The diagnosis was confirmed through computed tomography (CT) scan with oral contrast administration, highlighting the utility of this modality in diagnosing EPF. The patient was further referred to the surgical oncology team for esophageal resection and fistula closure.

## Introduction

Esophagopleural fistula (EPF) is a rare esophago-respiratory fistula characterized by a connection between the esophagus and the pleural space. In adults, it often follows surgery, malignancy, infection, trauma, or radiation therapy. Patients typically present with nonspecific respiratory symptoms: fever, acute dyspnea, productive cough, and lung consolidation and effusion. EPFs are more commonly detected on the right side due to anatomical proximity, with left-side occurrences being rare [1].

This case report underscores the importance of early diagnosis and multidisciplinary treatment in managing complex cases of EPF, particularly those caused by high-grade squamous cell carcinoma of the esophagus, and highlights diagnostic and therapeutic challenges.

## Case presentation

**Figure 1:**
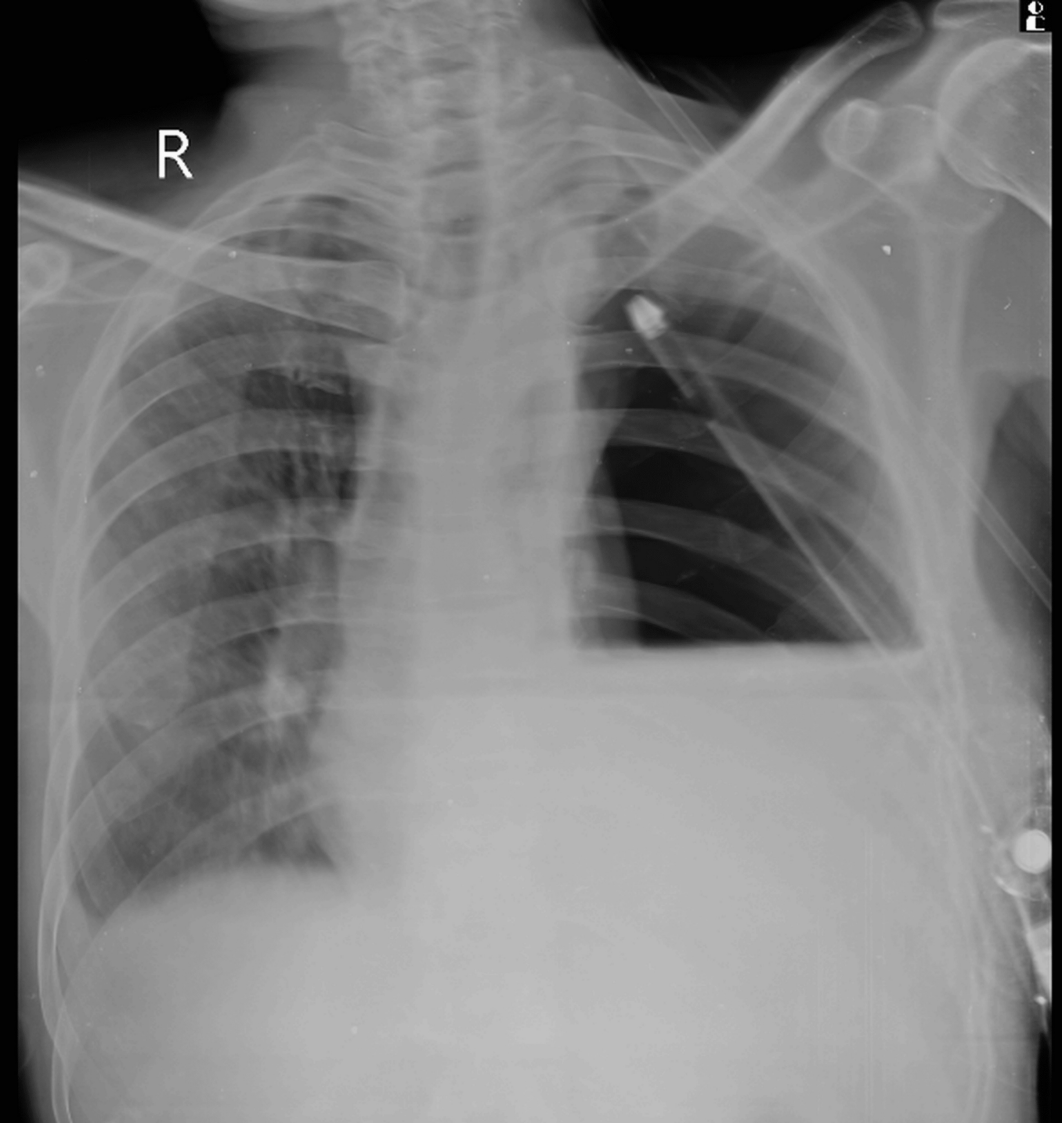
Chest X-ray suggestive of left lung collapse with hydropneumothorax with intercostal drain in situ

A 64-year-old male was referred to our center with a diagnosis of left-sided empyema with an intercostal drain in situ for further evaluation and management. The patient presented with a two-month history of cough, breathlessness, weight loss, loss of appetite, and chest discomfort. Notably, he had no history of smoking, major surgeries, or prior trauma. Upon examination, the patient appeared cachexic, and chest examination revealed an intercostal drain on the left side with no water column seen in the drain bag, left hemithorax reduced movements, dullness on percussion in the fifth intercostal space along the midclavicular, midaxillary, and seventh intercostal space in the left scapular line. There were also diminished breath sounds on the left infrascapular and infra-axillary areas. A chest X-ray showed a left-sided hydropneumothorax with a collapsed left lung and an intercostal drain in situ (Figure 1).

Laboratory tests indicated anemia and hypokalemia, while sputum and pleural fluid testing for acid-fast bacilli (AFB) were negative. Ultrasonography revealed a moderate left pleural collection with internal echoes suggestive of empyema.

**Figure 2:**
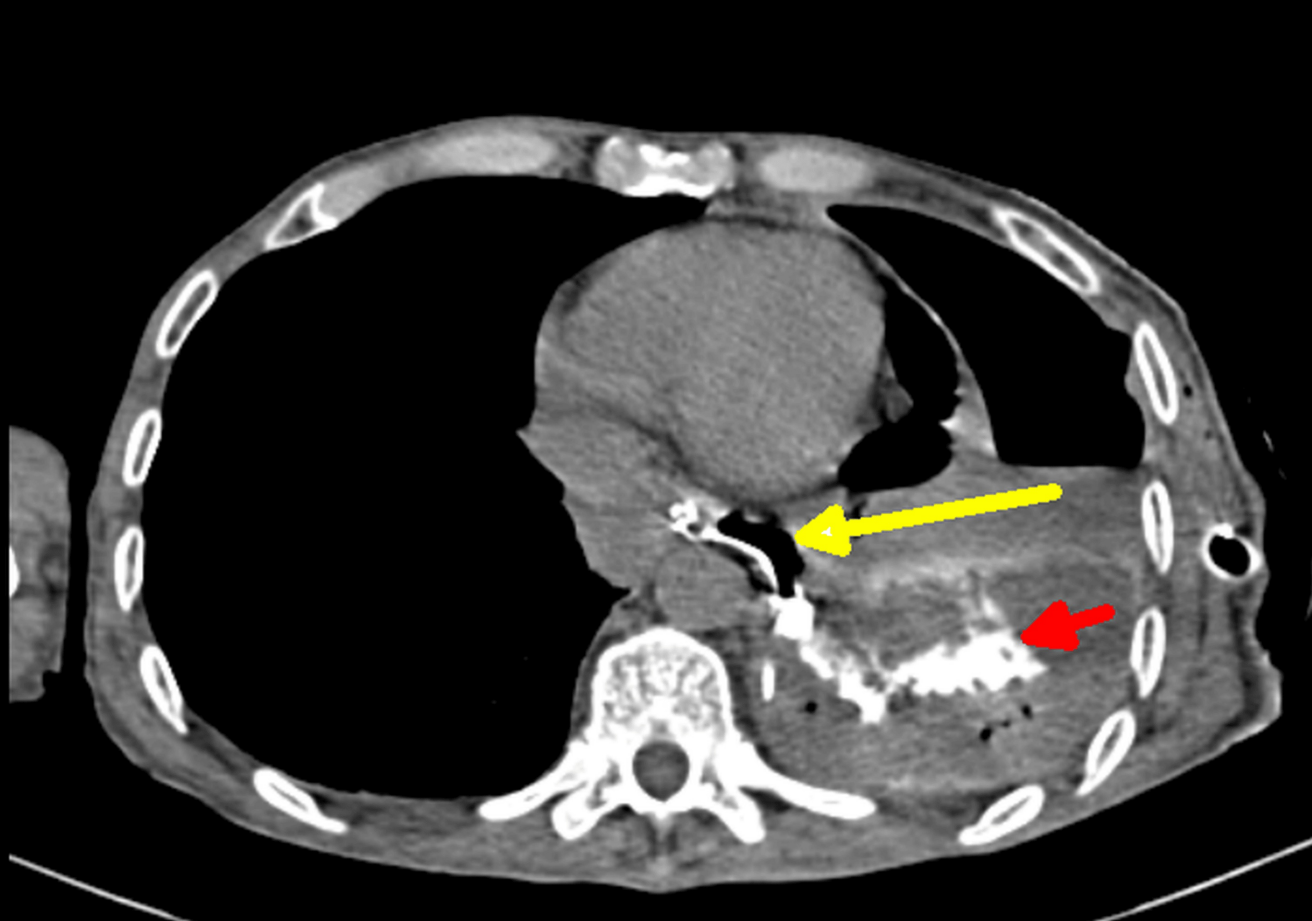
CT with oral contrast scan of thorax with oral contrast showing esophagopleural fistula tract (yellow arrow) and collection of oral contrast in the pleural cavity (red arrow) CT, computed tomography

The patient was admitted and started on intravenous antibiotics, correction of electrolytes, and nutritional support. Despite treatment over four to five days, it was observed that the patient had non-resolving empyema with excessive drain from the intercostal tube. CT scan of the thorax showed a moderate pleural collection with a collapsed left lung and the intercostal drain in place. Importantly, a linear fistulous communication from the distal esophagus was noted on imaging, confirmed by oral contrast administration, showing the contrast medium entering the pleural collection through the fistula tract, illuminating the pleural space (Figure 2). In all, 50 mL of diluted gastrografin oral contrast was used for imaging.

**Figure 3:**
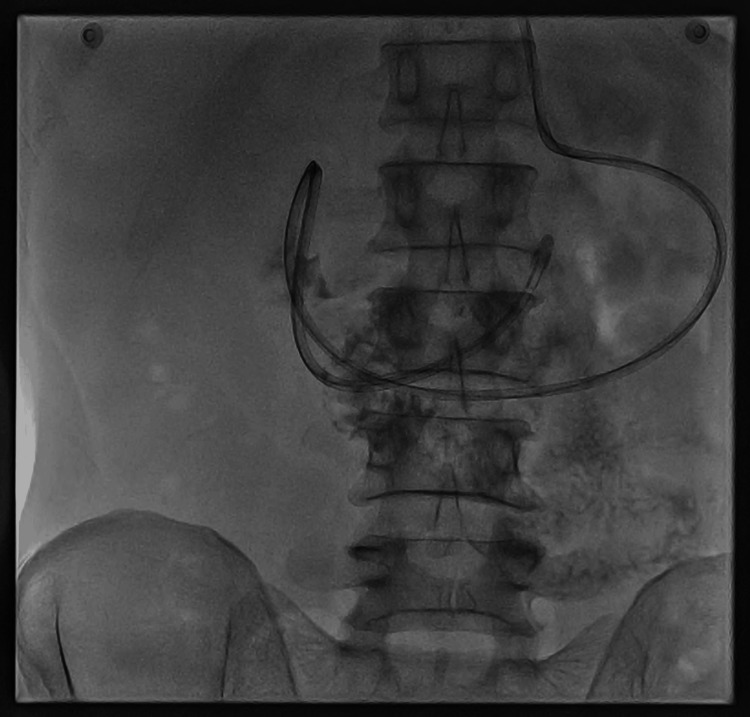
Fluoroscopy-guided nasojejunal tube insertion

A diagnosis of the esophageal-pleural fistula was established, prompting upper gastroscopy for visualization of the fistula and biopsy. The gastroscopy revealed irregular mucosa over the distal esophagus and a narrowing of the lumen; however, the fistula opening was not visualized. A biopsy was taken, and the results indicated high-grade squamous cell carcinoma of the esophagus. The patient underwent a fluoroscopy-guided nasojejunal tube placement for nutritional support (Figure 3).

The patient was further referred to surgical oncology for esophagus resection and fistula closure, but unfortunately, during the course of treatment, the patient experienced sudden hypotension and bradycardia, leading to his unfortunate demise.

## Discussion

ERFs, including tracheoesophageal and esophageal pulmonary fistulas, result from infections, inflammatory processes like Crohn’s and sarcoidosis, or iatrogenic causes [1]. Common cancers associated with ERF are those of the lung, esophagus, and mediastinum. EPF patients often present with sudden dyspnea, fever, chest tenderness, respiratory infection signs, and cavitating lesions with pleural fluid on radiographs [1]. Early diagnosis can be challenging due to nonspecific symptoms, but characteristic pleural fluid features like sour smell and food particles indicate esophageal communication [1].

Diagnosis relies on endoscopy (bronchoscopy and gastrointestinal endoscopy with methylene blue dye) or CT scans. Early diagnosis is crucial to prevent severe pulmonary complications, with contrast esophageal X-rays and oral contrast CT improving detection rates [2,3]. Imaging strategies include EGD, chest CT, and flexible bronchoscopy to identify fistulas [4]. Management involves addressing esophageal disruption and pyopneumothorax, with complications like sepsis and respiratory deterioration requiring individualized treatment, including thoracentesis, intravenous antibiotics, and nutritional support [5,6].

Existing literature points out the various clinical presentations of EPF symptoms ranging from high-grade fever, cough, and shortness of breath mimicking pneumonia to dyspnea, chest pain, and recurrent pneumonia, more so after thoracic intensity-modulated proton therapy. The EPF may present as a complication of surgery for esophageal cancer, systemic sclerosis, or even a sequela of esophageal stenting [7,8]. 

Cui et al. (2017) researched diagnostic accuracy in patients with EPF through upper gastroscopy [9]. Their study provided insight into clinical manifestations, management of EPF, and early recognition based on proper treatment strategies. They highlighted the effectiveness of diagnostic modalities like chest CT and contrast esophagography in diagnosing EPF with a high degree of accuracy through a comprehensive review of the related literature [9]. Moreover, the EPF in association with esophageal malignancy raises the importance of considering malignancy as one of the potential etiological factors in cases of EPF. Managing EPF concurrent with malignancy poses intricate treatment challenges. The cases may escalate to life-threatening scenarios, especially when malignancy is involved. Treatment modalities may include the endoscopic placement of stents, pseudo-aneurysm embolization, and esophageal stent graft placement. However, achieving closure of fistulas proves challenging, often requiring multiple interventions, such as polyglycolic acid sheets, fibrin glue, and vascular embolization plugs. While surgical options are available, for example, esophagectomies, they carry radical implications with the associated complications and mortality rates [10,11].

The management of EPF requires individualized, multidisciplinary care by surgery, pulmonology, gastroenterology, and oncology. Practice variation is based on characteristics such as the location of the fistula, the viability of the tissues, and the patient’s physiological condition. Individualized approaches, mainly consisting of endoscopic interventions, must be rapidly initiated, with surgical alternatives being offered in special circumstances only. Surgery should be done as early as possible in order to prevent pulmonary complications and improve patient outcomes. Endoscopic stenting for EPF closure results in substantially improved quality of life and survival [12,13].

## Conclusions

The case of esophageal fistula leading to pyopneumothorax represents a complex clinical scenario requiring prompt and precise intervention. This report highlights the importance of early diagnosis and a multidisciplinary approach to managing such complications. Esophageal disruption, when combined with pyopneumothorax, poses significant challenges due to the intricate anatomy and the risk of severe infection, severe malnutrition, and sepsis. Although our patient did not survive, the outcome for future cases is hinged on a comprehensive strategy involving intercostal drainage, aggressive antibiotic therapy, and nasojejunal tube insertion for nutritional support and further intervention by the surgical oncology team. This case highlights the critical need for vigilance in recognizing the symptoms of esophageal injury and the necessity for swift, coordinated care to optimize patient outcomes. Continued research and clinical awareness are essential to improve management protocols for similar cases in the future.
